# Effectivenes of incentive constraint policies in enhancing green bond credit rating and certification: A theoretical and empirical study

**DOI:** 10.1371/journal.pone.0289750

**Published:** 2023-11-16

**Authors:** Hanyi Zhao, Yixiang Tian, Xiangyun Zhou, Xuefeng Zhao

**Affiliations:** 1 School of Management and Economics, University of Electronic Science and Technology of China, Chengdu, Sichuan, China; 2 Shenzhen Institute of Information Technology, Shenzhen, Guangdong, China; 3 Jiangsu Ocean University, Lianyungang, Jiangsu, China; University of Almeria, SPAIN

## Abstract

This paper aims to effectively reduce CO2 emissions by examining the impact of three distinct incentive and constraint policies on the quality of rating and certification information in China’s green bond issuance market. To accomplish this, the government has implemented incentives, while regulators have introduced constraints to curb the spread of inflated rating and certification information. We build on the integrated rating and certification regulation mechanism by presenting a two-stage Stackelberg game model that involves four key participants: the China Securities Regulatory Commission, local governments, green evaluation and certification agencies, and credit rating agencies. We incorporate environmental effects indicators into the expected utility of rating and certification agencies to investigate the equilibrium conditions under three policy scenarios: a single financial incentive policy, a single regulatory constraint policy, and a combined incentive and constraint policy. The paper employs Stackelberg game theory to analyze how different policies mitigate the occurrence of “inflated” ratings and “greenwashing” in certifications. Numerical analysis is conducted to validate the theoretical findings. Moreover, we assess the impact of these policies on the quality of rating and evaluation information, using data from China’s green bond issuance market between 2016 and 2021. Our research offers valuable management insights and regulatory recommendations for both regulators and local governments.

## 1. Introduction

**Fig 1:**
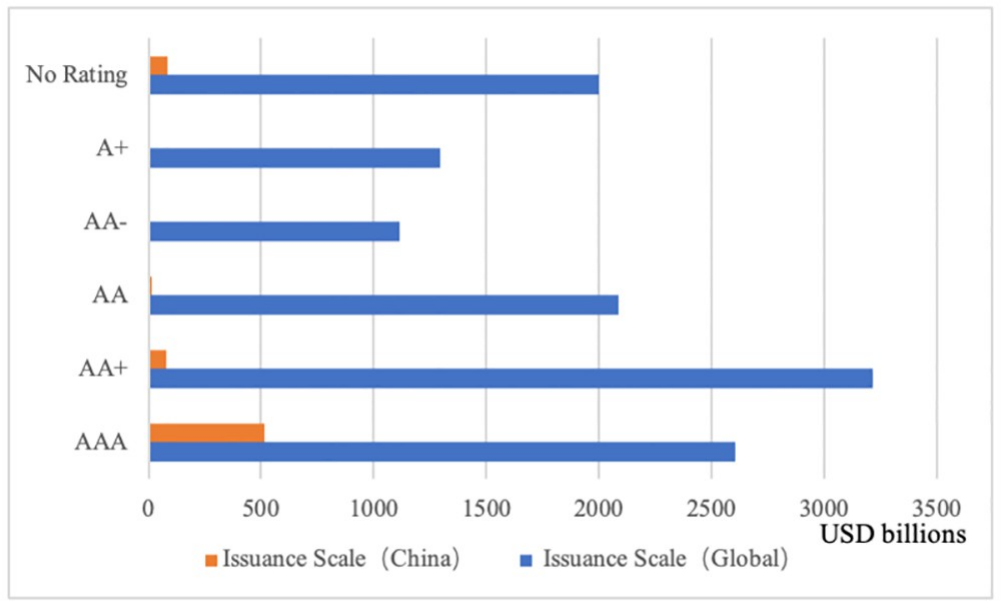
Credit rating distribution of global and Chinese Green Bonds in 2021. Data source: Climate Bonds Initiative.

Finance instruments can play a vital role in addressing climate change by directing funds towards environmentally-friendly projects and promoting industrial upgrading. As one of the fastest growing components of China’s green financing system, green bonds distinguish themselves from general credit bonds by funding sustainable development projects with high green economic benefits [1]. And it has been effective in channeling privately offered funds and bond market capital towards green projects [2]. However, the green bond market faces the reputational risk of bond “greenwashing” and the default risk of “inflated” ratings, as weak regulation and inflated financial incentives create opportunities for risky behavior in rating and certification [3, 4]. From 2016 to 2022, statistics from the Wind database indicate that 99.22% of corporate green bonds issued held credit ratings of AA or higher. Among them, AAA-rated green bonds represented 89% of total issuances in 2021, with fewer low-rated bonds. As is shown in Fig 1, comparing 2021 global green bond credit ratings, China’s AAA-rated green bonds proportion is higher than the global average, indicating that Chinese green bonds generally have higher ratings [3, 4]. According to Lianhe Credit Rating Co., Ltd, China issued a total of 485 green bonds in 2021, amounting to CNY 6,075.42 billion. Among these, CNY 1,772.13 billion were not explicitly allocated to specific sectors. Out of 311 green bonds with clearly defined fund usage, CNY 279.27 billion were used for non-green purposes, accounting for approximately 8.32% of the total. This significantly exceeds the Climate Initiatives Organization’s standard, which requires that “the proportion of non-green usage should not exceed 5%.” Furthermore, evaluations of environmental effects do not constitute a significant portion of the green bond rating system in China. This results in a problem where bond ratings are unable to accurately reflect variations in environmental impacts [5].

These risks intensify uncertainty surrounding the utilization of green bonds. Owing to the pronounced policy guidance features of green bonds, a crucial practical challenge in the development of China’s green bond market is determining how to amplify the policy impact to minimizing the credit and default risks associated with green bonds [6]. In this research,we explores the effect of different incentive and constraint policy mechanisms on the quality of green bond rating and certification information from both theoretical and empirical aspects, which can provide a solid theoretical foundation and practical guidance for the growth of China’s green bond rating evaluation industry.

Financial incentives and regulatory constraints are vital for green bond rating and certifications. According to both the *the Green Bond Operation Report of China* and Lianhe Credit rating Co., Ltd, a total of 29 provinces and cities in China have issued incentive policies about green bonds from 2017 to 2022, including preferential issuance conditions and government financial subsidies. The analysis of *the effectiveness of local government green financial policies* show a positive correlation between green bond issuance scale and financial policy implementation [7]. However,under the issuer-pays system, excessive incentives may lead to “greenwashing” by encouraging issuers to collaborate with rating agencies for higher ratings and lower financing costs [8]. These policies establish an interactive relationship between governments and participants of green bonds market.

To maintain stability in the green bond certification market, regulatory constraints should balance incentive policies. Some researchers argue that combining regulatory incentives and constraints enhances efficiency [9, 10]. [11] compared the quality of China’s bond rating information from the perspective of asymmetric regulation, and believed that strengthening regulation can effectively solve the problem of rating overestimation. The research of [12] shows that the strengthening of the regulation of the rating system and the dissatisfaction of investors with false rating information will prompt rating agencies to provide more conservative ratings and reduce the situation of inflated ratings. [13] noted that compared to separate policy incentives or regulatory penalties, a double mechanism of government incentives and penalty constraints can help alleviate the government’s financial pressure. This approach is more efficient in achieving an optimal combination of benefits and balance between supply and demand sides. According to the above research, how to optimize regulation through the harmonization of regulatory incentives and constraints is essential for effective regulation.

**Fig 2:**
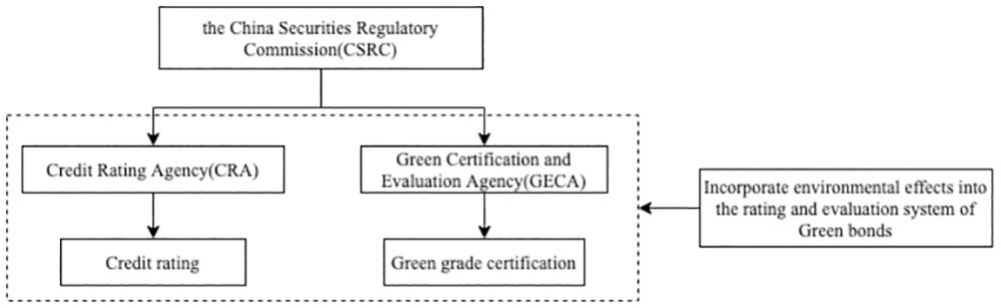
Integrated rating and certification regulation structure.

China’s “double rating + double regulation” mechanism for green bond issuance is a complex system involving the China Securities Regulatory Commission (CSRC), Green Bond Committee(GBC), credit rating agencies (CRAs), and green certification and evaluation agencies (GECAs) [14]. Under the double regulatory system, different types of green bonds have different regulatory requirements and legal norms, which is easy to cause duplication of regulation and vacancies in regulation [15]. In addition, the cost of regulation is high and the probability of inspection is low, which lead to an imperfect regulation mechanism [16]. To address these issues, the State Council of China introduced the “Guiding Opinions on Accelerating the Establishment of a Green, Low-Carbon, and Circular Development Economic System” (*Green System Guidelines*) in Feburary 2, 2021, asked to providing clear and uniform standards for green financing projects, fund utilization, and bond duration information disclosure, which would help streamline regulations across various green bond types in China. Meanwhile, the policy suggests enhancing fiscal and taxation support for green finance in order to promote its robust development. [17] proposed an “integrated rating and certification regulation mechanism(integrated regulatory mechanism)” for green bonds. This mechanism allows green assessment agencies and credit rating agencies to perform certifications both before and during the bond’s duration, all under the regulation of a unified regulatory body, the structure of integrated regulatory mechanism is shown in Fig 2. As China’s green bond market regulations become increasingly standardized, it is crucial to investigate the impact of incentives and constraints on the quality of green bond rating and certification within integrated regulatory mechanism.

Under the integrated regulatory mechanism, there are multiple interacting participants in the green bond rating and certification market. Game theory has proven to be an effective method for modeling and analyzing the interaction between policymaker and participants of green finance market. [18] developed an evolutionary game model with the government, financial institutions, enterprises, and consumers as participants, examining the impact of various parameters on the development of the green finance market and highlighting the need for stronger government regulation. [14] conducted research on the effectiveness of the double rating mechanism and the integrated rating mechanism with Hotelling game model, taking into account the spatial effect of green bonds. According to [19], because that the government typically holds more power than GSC, making the Stackelberg game a fitting model to describe the interactions. They use a Stackelberg game to evaluate the interaction between the government and the Green Supply Chain(GSC), and the effects of three carbon regulation policies on GSC are studied. [20] analyzed a multi-stage Stackelberg model for sustainable production inventory, incorporating both a manufacturer (leader) and a retailer (follower). [21] explored a two-period procurement decision of green supply chain participants under a manufacturer-Stackelberg game, comparing the profits, greening level, and environmental improvement of each member under different incentive policies.

This paper examines the effects of three incentive and constraint policies on the quality of rating and certification information in China’s green bond issuance market within an integrated regulatory mechanism. Taking into account the sequence between policy announcements and the strategies of rating and certification agencies, a two-stage Stackelberg game is developed, involving GECAs, CRAs, the CSRC and local governments,and incorporating environmental effects indicator into rating and certification agencies’ expected utility. We analyzes the equilibrium conditions of two agencies acorss nine game scenarios under three different incentive and constraint mechanism: single financial incentive policy, single regulatory constraint policy and coexisting incentive and constraint policy. Theoretical analysis and simulation compares strategic influences and optimal expected profit under different policies, while an empirical test utilizes an ordered Logit model on China’s green bond data from 2016 to 2021 to assess the impact of the three policies on green bond rating and certification information quality, which is conducted to validate the theoretical findings. The study proposes incentive and constraint policy options at various stages to optimize green bond rating and certification information quality from a regulatory perspective, based on theoretical and empirical findings.

The innovations of this study are as follows: (1)The current regulatory mechanism for green bonds in China is characterized by a double regulatory approach that is steadily transitioning towards an integrated regulatory mechanism. Existing literature on China’s green bond regulation primarily focuses on the context of multiple regulatory frameworks, leading to a constrained research perspective and background. In contrast, our study embraces the official concept of integrated regulatory as its research background, offering a novel, forward-looking perspective on the topic. (2) Existing research [10, 11, 13, 22, 23] on the interaction between regulatory policy and rating quality often utilizes game models without accounting for the asymmetry in the decision-making sequence between policy issuance and rating evaluation behavior. Referring to the interaction between policymakers and GSCs in these study [19, 20], our study constructs a two-stage Stackelberg game model that considers the action sequence for four participants, more accurately representing the impact of incentive and constraint policies on rating and certification information, resulting in more reasonable theoretical conclusions. (3)Current research [6, 9, 14, 20, 21, 24] primarily focuses on the theoretical analysis of incentive and constraint mechanisms’ impact on green products, with limited empirical research to support the theoretical findings. Our study manually compiles policy-related data, gathers green bond data from multiple databases, and processes raw data using a multi-category approach. We conduct an empirical test using the ordered Logit model to assess the effectiveness of various incentive and constraint policies, which bolsters the credibility of our theoretical conclusions.

The rest of the paper is organized as follow. Section 2 introduces the Stackelberg model. Section 3 provides the available conditions of single incentive policy, single regulatory constraint policy and coexisting incentive constraint policy under the integrated regulatory mechanism to prevent “inflated” rating and “greenwashing” evaluation. Section 4 shows the numerical analysis and the simulations under three different policies mechanism. Section 5 is an empirical analysis under the three different policies in China. Section 6 makes conclusions and research prospects. The mathematical proofs, tables of the empirical analysis are shown in the S1 Appendix.

## 2. The model

### 2.1 Problem description

Incentive and constraint policies are usually enacted by governments and regulators. For simplicity, this study focuses on four principal participants: the CSRC, Local Governments, GECAs, and CRAs. To investigate the effects of government financial incentives, CSRC regulatory constraints, and environmental benefits of green bond financing projects on the green bond rating and certification process, a two-stage Stackelberg game model is employed, involving the government, the CSRC, and the rating and certification agencies.

In this model, the sequence of the game is a strategic component. In the first stage, the local government and the CSRC, acting as leaders, decide whether to provide financial subsidies and impose regulatory constraints for successful green bond issuance. Their leadership status affords them an edge, allowing them to shape the follower’s options by moving first. The CSRC and local governments anticipate reactions from rating and certification agencies when they set the regulatory and incentive coefficients.

In the second stage, the GECAs and CRAs, having complete information about the leaders’ decisions, select the green certification level and the credit rating level. They can make more informed decisions, using the leaders’ first moves to optimize their responses. This approach allows green bond issuers to indirectly influence rating and certification results generated by rating agencies through expected returns from local government incentives. Concurrently, investors indirectly impact rating agencies’ reputation through local governments’ recognition and penalties imposed by the CSRC.

**Fig 3:**
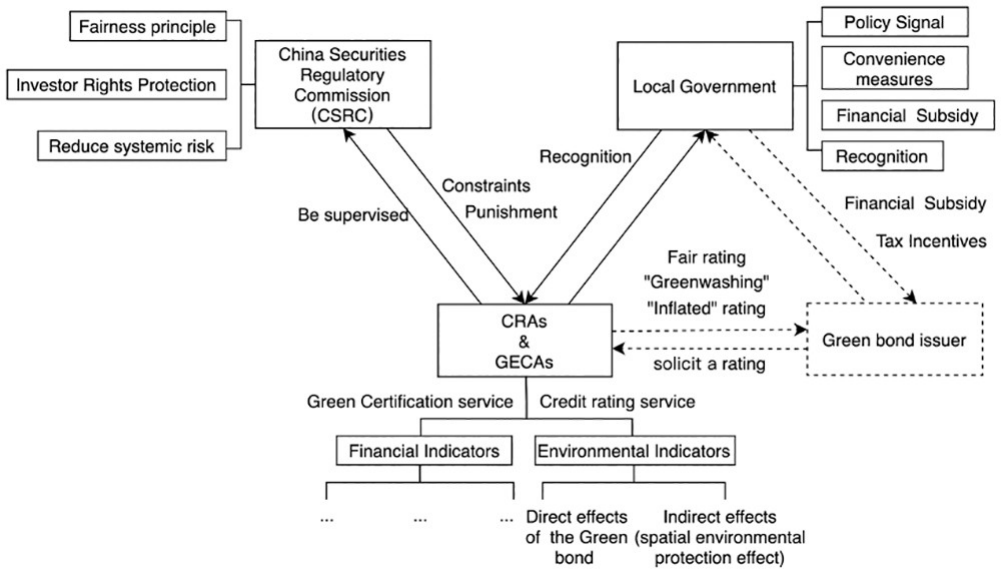
The integrated rating and certification regulation mechanism in China’s green bond issuance market.

Therefore, this strategic order of actions within the model doesn’t necessarily denote any temporal precedence, but it serves as a mechanism to simulate strategic advantages based on information availability and decision-making influence. The structure of the model is presented in Fig 3.

### 2.2 Assumptions

There is no substitutability between CRAs and GECAs. We consider that there are two types of green bonds *j* = {*G*, *B*} provided by bond issuers and two types of agencies *i* = {*E*, *C*} that GECAs evaluate the financing project’s environmental benefits and CRAs rate the financial default and solvency. Now, referring to the studies of [11, 25], the following assumptions are made to establish the framework for the forthcoming mathematical models:

**Assumption 1** Consider a two-stage game between players. The CSRC and Local Governments are assumed to be the two leader players, while GECAs and CRAs are considered to be the follower players. The CSRC can choose to impose regulatory constraints or not, and Local Governments can choose the type of incentive policies. The GECAs and CRAs pursue the best response strategies against the decisions made by the governments and CSRC. The analysis of the hierarchical game between the leaders and followers is performed using the backward induction method. All four players in the game are assumed to be risk-neutral.

**Assumption 2** There are two types of green bonds in the green bond issuance market, high-quality bonds *G* and low-quality bonds *B*, namely *m* = {*G*, *B*}. The high-quality bonds referred to in the article refer to green bonds that contribute high finance and environmental benefits to the invested projects and have a low probability of financial/environmental default. Assume that the total amount of green bonds issuance is 1, the amount of bonds G in the green bond issuance market is λ, and the amount of bonds B is 1 − λ.

**Assumption 3** In the first stage, the local government has strategies set *w*_*i*_, That is, whether to issue relevant incentive policies to two agencies. It aims to maximize the environmental benefits of green bonds in the region, that is, to minimize carbon emission reductions. If local government choose to issue incentive policies, thus the government low-carbon subsidy obtained by GECAs and CRAs is $\left\{ \begin{array}{c} r_{m_{1}}=k_{1}\left(g_{m}-g\right),g_{m}>g\\ 0,g_{m}\leq g \end{array}\right.$, *k*_1_ is the adjustment factor of the unit incentive coefficient (Wang,2009). *g* is the lowest limit of the green grade of green bond financing projects, and local government subsidies for green bonds are positively correlated with environmental benefits. Local government promulgating incentive policies in stage 1 will incur a policy publication fee of *C*_*L*_.

**Assumption 4** In the first stage, CSRC’s choice of a rating regulatory strategy *z*_*i*_ during this period is whether regulators issue strict restrictive policies for CRAs and GECAs to maximize the social utility (For simplicity, we consider maximizing regulatory efficiency). If CSRC issues strict constraint policies for CRAs or GECAs in this period, regulators promulgating strict binding policies in stage 1 will incur a policy publication fee of $C_{A}.\{\begin{array}{c} r_{m2}=k_{2}\left(d_{m}-d\right),d_{m}>d\\ 0,d_{m}\leq n \end{array}$, *k*_2_ is the adjustment factor of the unit constraint coefficient.

**Assumption 5** In the second stage, the decision set *e*_*i*_ and *v*_*i*_ of each agency (GECAs and CRAs) is about whether fair certification/rating or not. When the local government and CSRC pursues an incentive/constraint policy, they should make the corresponding decisions to maximize their expected profits. The certification/rating fee *f*_*i*_ is set by GECAs and CRAs. *f*_*i*_ is correlated with *r*_*m*1_ and the utility of issuers *u*_*m*_. When agencies give the “greenwashing” or “inflated” rating, *f*_*i*_(*u*_*m*_, *r*_*m*_) = (1 + *r*_*m*1_*u*_*m*_)*f*_*i*_, and *f*_*i*_(*r*_*m*_, *u*_*m*_) = *f*_*i*_ when they choose fair strategy. Except these, the key factor for green bonds is environmental benefits, and this factor have different weight in green certification and credit rating.

**Assumption 6** Agencies collect information and give the final green and credit grade to bonds *m* ∈ *g*, *b*. The quality of information obtained by agencies is *θ*_*i*_ ∈ *g*, *b*. *D*_*ij*_ is the demand function for bond issuers to purchase green and credit grade provided by agencies i. The utility of issuers is *u*_*j*_. According to [26], there are three scenarios of grade issuance (assumed that there will be no undervaluation):

$$\left\{ \begin{array}{c} D_{i}=\lambda_{i};\theta_{i}=g,m=G,u_{m}>f_{i}\\ 1-\lambda_{i};\theta_{i}=b,m=G,u_{m}>\left(1+r_{m1}u_{m}\right)f_{i}\\ 1-\lambda_{i}2;\theta_{i}=b,m=B,others \end{array}\right.$$

**Assumption 7** According to [27], we capture the spatial effect *E*_*m*_ of green bonds as environmental benefits, then let *p*_*i*_ ∈ (0, 1) be the different weight in two agencies, *p*_*G*_ > *p*_*C*_. Referring to the research of [28], it is assumed that environmental rating/certification input cost have a quadratic relationship with environment effect, that is, the environmental rating/certification input cost is $\frac{\lambda_{i}p_{i}}{2}E_{m}^{2}$.

**Assumption 8** It is possible that GECAs and CRAs may collude based on the interests of the bond issuer. We assume that the probability of collusion between the rating agencies is *γ*, where the value is 1 if the two agencies collude and 0 otherwise. In the green bond issuance market, the green grade and credit grade are interdependent. We assume that when “greenwashing” occurs, “inflated” rating will inevitably follow, but not necessarily vice versa, which implies that there are not necessarily any single “inflated” rating scenarios.

**Assumption 9** In the second stage, agencies may conspire to provide “inflated” rating or “greenwashing” evaluation in order to gain more profits. The CSRC will impose corresponding penalties *C*_*s*_ with these two behaviors by GECAs and CRAs, *σ*_*RC*_ ∈ (0, 1) is the regulatory discount factor [25]. Based on the strategy of CRSC in stage 1, the *C*_*s*_ will be:

$$C_{s}=\{\begin{array}{c} \sigma_{RC}r_{m_{2}}(1-p_{i}\cdot \;\text{ln}\;(E_{m}))(1+r_{m1}u_{m})f_{i},z_{i}=1\\ \sigma_{RC}(1+r_{m1}u_{m})f_{i},z_{i}=0 \end{array}$$

**Fig 4:**
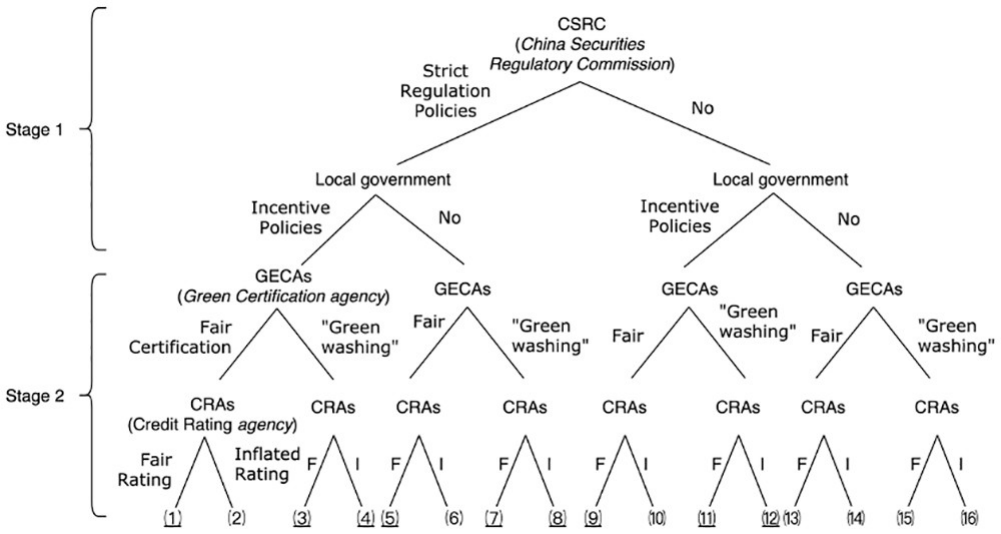
Two-stage game structure.

Fig 4 presents the structure of the two-stage game, which encompasses twelve scenarios. Based on the description and assumptions of this two-stage game, the problem is formulated in eight scenarios, which are highlighted.

### 2.3 The expected utility of CRAs and GECAs(Stage 2)

The expected profits function of rating and certification agencies constructed by [22] is as follows:

$$\begin{array}{c} {\bar{R}}_{RA}=(1-\sigma_{RA})\sum_{t=1}^{\infty }\sigma_{RA}^{t-1}\cdot D_{i}^{t}\cdot f_{i}^{t} \end{array}$$ (1)

The investor discount factor is represented by *σ*_*RA*_ [22]. This model does not account for environmental benefits, local government incentive policies, or green bond regulatory constraints. Therefore, our initial step is to incorporate the impact of environmental benefits on the validity of rating and certification information into the expected return function for rating and certification agencies [27]. We utilize two factors, *r*_*m*1_ and *r*_*m*2_, to illustrate the influence of incentives and constraints coefficients [11]. These three modifications aim to provide a more accurate analysis of the applicability of the local government and CSRC’s incentive and constraint policies. As per Eq (1), the expected return of optimized agencies, denoted as *i* = *E*, *C*, is presented in Eq (2):

$$\begin{array}{c} R_{i}=[1-r_{m_{1}}(1+p_{i}E_{m})]\cdot D_{i}\cdot f_{i}(u_{m},w_{i})-\frac{\lambda_{i}p_{i}}{2}E_{m}^{2}-\gamma \cdot C_{s}(z_{i}) \end{array}$$ (2)

*R*_*i*_ is the improved expected profits of two agencies *i* = {*E*, *C*}. The remaining variables are defined in Secion 2.2.

### 2.4 The expected utility of local government(Stage 1)

The objective of local governments is to maximize social welfare, which encompasses environmental protection and economic growth, among other factors [23]. This study focuses on green bonds and defines the target function of the local government as the minimization of carbon emissions from local green bonds, considering the maximization of local government environmental benefits. The environmental benefits of local governments can be viewed as a trade-off between bond issuers, rating agencies, and the utility function of environmental benefits [24]. For simplicity, we define the objective function of local governments as the total of economic and environmental benefits derived from bond issuance, subtracting the costs associated with policy announcement and implementation. The objective function of the government is presented in Eq (3):

$$\begin{array}{c} U_{gov}=(1+p_{i}E_{m})R_{i}-\sum_{t=1}^{\infty }w_{i}\cdot (1-p_{i}E_{m})\cdot C_{L} \end{array}$$ (3)

Assume that environmental benefits *E*_*m*_ are the total carbon emissions cut by green bond projects in an area. We also assume that government costs mainly come from the expenses of setting up and carrying out the incentive policies chosen in the first stage. The remaining variables are defined in Secion 2.2.

### 2.5 The expected utility of CSRC (Stage 1)

The expected profits function of regulatory constructed by [25] is as follows:

$$\begin{array}{c} {\bar{c}}_{RE}=(1-\sigma_{RE})\sum_{t=1}^{\infty }\sigma_{RE}^{t-1}\cdot (c_{A}\cdot \sum_{i=1}^{n}z_{i}^{t}+c_{M}\cdot \sum_{i=1}^{n}W_{i}^{t}) \end{array}$$ (4)

According to [11, 25] and Section 2.2, *σ*_*RE*_ represented the regulatory discount factor,$W_{i}^{t}$ represents whether agencies qualify for the provision of rating or certification information. If agencies *i* can issue ratings, $w_{i}^{t}=1$; Otherwise, $w_{i}^{t}=0$. Besides, every agencies have the regulatory cost *C*_*M*_ in period t. The goal of the CSRC is to enhance the efficiency of green bond regulation, meaning to maximize the benefits of regulation while minimizing costs. The regulatory efficiency is dependent on the regulatory capacity of the department and is represented by the balance between the benefits and costs of regulation [29]. This study uses the cost-benefit theory under the RDRs regulatory framework to optimize the profit function of the China Securities Regulatory Commission and the cost function constructed by [25]. The objective function for maximizing regulatory efficiency is shown in Eq (5):

$$\begin{array}{c} maxU_{csrc}=max\left(R_{csrc}-C_{csrc}\right) \end{array}$$ (5)

Among them, the revenue of CSRC constructed based on the source of revenue:

$$\begin{array}{c} R_{csrc}=C_{s}\left(z_{i}\right) \end{array}$$ (6)

For simplicity, this section presumes that all rating and evaluation agencies possess the necessary business qualifications. Additionally, we assumed that regulatory costs primarily arise from the issuance and enforcement of regulatory constraints chosen in the first stage. The improved *C*_*CSRC*_ based on the Eq (4) constructed by [25] is as follow:

$$\begin{array}{c} C_{CSRC}=(1-p_{i}E_{m})\cdot C_{A}\cdot \sum_{t=1}^{\infty }z_{i} \end{array}$$ (7)

The specific components of Eq (5) are constituted by Eqs (6) and (7). Assume that $\frac{dR_{CSRC}}{dC_{A}}>0,\frac{d^{2}R_{CSRC}}{dC_{A}{}^{2}}<0,\frac{dC_{CSRC}}{dC_{A}}>0,\frac{d^{2}C_{CSRC}}{dC_{A}{}^{2}}>0$. This suggests that with heightened regulatory levels, regulatory benefits decrease as regulatory costs escalate. For simplification, we assume that the CSRC’s revenue primarily originates from penalties imposed on agency violations.

## 3. Solution approach

### 3.1 Under both incentive policies and constraints

#### Scenario 1:Fair strategies

When both two agencies choose fair strategies,according to Eq (2) and Section 2.2, the expected profits of two agencies are as follow:

$$\begin{array}{c} R_{i}=[1+r_{m_{1}}(p_{i}E_{m}-1)]\cdot (\lambda_{i}+\frac{1-\lambda_{i}}{2})f_{i}-\frac{\lambda_{i}p_{i}}{2}E_{m}^{2} \end{array}$$ (8)

**Proposition 1** Under both incentive policies and regulatory constraints, when condition $r_{m_{1}}\in [\frac{2\lambda_{i}E_{m}}{f_{i}(1+\lambda_{i})},1]$ is satisfied, GECAs and CRAs are likely to adopt fair rating and certification strategies. The value of $r_{m_{1}}$ is positively related to *E*_*m*_. When the environmental impact is stronger, the financial incentives provided by the Local Governments are greater, leading the two agencies to be more inclined towards a fair strategy. For proof 1, please see S1 Appendix.

According to these values, we obtained lemma 1:

**Lemma 1** When the government’s subsidies are certain, and GECAs and CRAs adopt fair strategies under both incentive policies and constraints, the utility of GECAs and CRAs increases as the carbon environmental effect *E*_*m*_ of the financing project increases. The level of environmental effect is also impacted by incentives, with higher incentives $r_{m_{1}}$ resulting in a greater environmental effect.

#### Scenario 2:Fair certification and “Inflated” rating

In this scenario, the expected profits of two agencies are as follow:

$$\begin{array}{c} R_{geca}=[1+r_{m_{1}}(p_{G}E_{m}-1)]\cdot (\lambda_{i}+\frac{1-\lambda_{i}}{2})f_{i}-\frac{\lambda_{i}p_{G}}{2}E_{m}^{2} \end{array}$$ (9)

$$\begin{array}{c} \begin{array}{c} R_{cra}=(1-\lambda_{i})\cdot (1+r_{m1}u_{m})f_{i}-\frac{\lambda_{i}p_{i}}{2}E_{m}^{2}-\sigma_{RC}r_{m_{2}}\\ (1-p_{C}\cdot \;\text{ln}\;(E_{m}))(1+r_{m1}u_{m})f_{i} \end{array} \end{array}$$ (10)

**Proposition 2** Under both incentives and constraints, the strategy choice of CRAs is influenced by low-carbon subsidies offered by the government and the regulatory penalty rate. The risk of collusion between the GECAs and CRAs can be decreased when $r_{m_{1}}\in [\frac{2{\lambda_{i}E}_{m}}{f_{i}\left(1+\lambda_{i}\right)},1]$ and $r_{m_{2}}\in \left[\frac{1{-\lambda }_{i}}{\sigma_{RC}\left(p_{C}ln(E_{m})-1\right)},1\right]$. The proof 2 is in S1 Appendix.

#### Scenario 3:“Greenwashing” and Fair rating

In this scenario, the expected profits of two agencies are as follow:

$$\begin{array}{c} \begin{array}{c} R_{geca}=(1-\lambda_{i})\cdot (1+r_{m1}u_{m})f_{i}-\frac{\lambda_{i}p_{i}}{2}E_{m}^{2}-\sigma_{RC}r_{m_{2}}\\ (1-p_{C}\cdot \;\text{ln}\;(E_{m}))(1++r_{m1}u_{m})f_{i} \end{array} \end{array}$$ (11)

$$\begin{array}{c} \begin{array}{c} R_{cra}=[1-r_{m_{1}}(1-p_{G}E_{m})]\cdot (\lambda_{i}+\frac{1-\lambda_{i}}{2})f_{i}-\frac{\lambda_{i}p_{G}}{2}E_{m}^{2} \end{array} \end{array}$$ (12)

**Proposition 3** Under the coexisting incentive and constraint mechanism, the strategy choice of GECAs is influenced by the low-carbon subsidies offered by the government and the regulatory penalty rate. The risk of collusion between GECAs and CRAs can be reduced when $r_{m_{1}}$ falls within the range $\left[\frac{2\lambda_{i}E_{m}}{f_{i}(1+\lambda_{i})},1\right]$ and $r_{m_{2}}$ falls within the range $\left[\frac{1-\lambda_{i}}{\sigma_{RC}(p_{C}\;\text{ln}\;(E_{m})-1)},1\right]$. The proof 3 is in S1 Appendix.

From scenario 2 and scenario 3, combining with Proposition 1, we obtained the lemma 2:

**Lemma 2** The strategy choices of GECAs and CRAs are influenced by two factors: the environmental effect (*E*_*m*_) and the different weight (*p*_*i*_). A higher weight of environmental effects in GECAs and CRAs will result in more effective incentives and constraints. Given limited financial incentives, the CSRC and local government should prioritize increasing the environmental effect weights of the two agencies to increase the likelihood of fair strategies.

#### Scenario 4:“Greenwashing” and “Inflated” rating

Under this scenario, we assumed that two agencies will collude to maximum their own profit. The expect profits of two agencies is as follow:

$$\begin{array}{c} \begin{array}{c} R_{i}=(1-\lambda_{i})\cdot (1+r_{m1}u_{m})f_{i}-\frac{\lambda_{i}p_{i}}{2}E_{m}^{2}-\sigma_{RC}r_{m_{2}}\\ \left(1-p_{i}\cdot \;\text{ln}\;(E_{m}))(1+r_{m1}u_{m}\right) \end{array} \end{array}$$ (13)

**Proposition 4** Under the coexisting incentive and constraint mechanism, when the incentives and constraints are fixed, GECAs and CRAs are more likely to produce “greenwashing” certification or “inflated” rating results when $E_{m}\in [{\left(\frac{\sigma_{RC}r_{m_{2}}(1+u_{m}r_{m_{1}})f_{i}}{\lambda_{i}}\right)}^{\frac{1}{2}},\frac{1}{p_{i}})$. The proof 4 is in S1 Appendix.

According to the analysis of these four scenarios, we can obtain the lemma 3:

**Lemma 3** Under a coexisting incentive and constraint mechanism, the CSRC can enhance the penalties for “inflated” ratings or “greenwashing” certification by increasing the regulatory penalty rate. Meanwhile, the local government can improve the accuracy of green bond ratings and certifications by either increasing low-carbon subsidies or the proportion of environmental effects in the rating process, thus reducing the cost of finance incentives.

### 3.2 Under the strict regulation policies

#### Scenario 5:Fair strategies

Under the strict regulation mechanism, when both two agencies choose fair strategies, the expected profits of two agencies are as follow:

$$\begin{array}{c} \begin{array}{c} R_{i}=D_{i}\cdot f_{i}(u_{m},w_{i})-\frac{\lambda_{i}p_{i}}{2}E_{m}^{2}=(\lambda_{i}+\frac{1-\lambda_{i}}{2})f_{i}-\frac{\lambda_{i}p_{i}}{2}E_{m}^{2} \end{array} \end{array}$$ (14)

**Proposition 5** Under the strict regulation mechanism, without the low-carbon subsidies from local government, *E*_*m*_ is the most important factor to satisfy $E_{m}\geq {\left(\frac{1}{p_{i}}f_{i}\right)}^{\frac{1}{2}}$ for two agencies to ensure the fairness of the rating results. The proof 5 is in S1 Appendix.

#### Scenario 6:Fair certification and “Inflated” rating

In this scenario, the expected profits of two agencies are as follow:

$$\begin{array}{c} \begin{array}{c} R_{geca}=(\lambda_{i}+\frac{1-\lambda_{i}}{2})f_{i}-\frac{\lambda_{i}p_{G}}{2}E_{m}^{2} \end{array} \end{array}$$ (15)

$$\begin{array}{c} \begin{array}{c} R_{cra}=(1-\lambda_{i})f_{i}-\frac{\lambda_{i}p_{C}}{2}E_{m}^{2}-\sigma_{RC}r_{m_{2}}(1-p_{C}\;\text{ln}\;(E_{m}))f_{i} \end{array} \end{array}$$ (16)

**Proposition 6** Under the strict regulation mechanism, the probability of an “inflated” rating by the CRAs depends on the level of the regulation penalty rate *r*_*m*2_. When $r_{m2}\in [\frac{\lambda_{i}}{\sigma_{RC}f_{i}}E_{m}^{2},1]$, regulators can choose a single regulation mechanism to guide rating agencies to issue accurate rating information. The proof 6 is in S1 Appendix.

The derivation process is the same for scenario 7. According to the above comparison, we can get lemma 4:

**Lemma 4** The regulatory penalty rate under this mechanism is higher than that under the incentive and constraint, and the CSRC needs higher regulatory penalties to ensure that CRAs issue fair rating information. Higher regulatory penalty means higher regulatory cost. It can be seen that when $r_{m2}\in [\frac{1-\lambda_{i}}{\sigma_{RC}(p_{C}\;\text{ln}\;(E_{m})-1)},\frac{\lambda_{i}}{\sigma_{RC}f_{i}}E_{m}^{2}]$, the regulatory cost of the CSRC under the coexisting incentive and constraint policies is lower.

#### Scenario 8:“Greenwashing” and “Inflated” rating

In this scenario, the expected profit of two agencies is as follow:

$$\begin{array}{c} \begin{array}{c} R_{i}=(1-\lambda_{i})f_{i}-\frac{\lambda_{i}p_{i}}{2}E_{m}^{2}-\sigma_{RC}r_{m_{2}}(1-p_{i}\;\text{ln}\;(E_{m}))f_{i} \end{array} \end{array}$$ (17)

Comparing the regulatory penalty rate between Scenario 4 and Scenario 8, it can be observed that when both the green certification agency and the credit rating agency adopt an unfair certification strategy, we can obtain $\frac{\lambda_{i}}{\sigma_{RC}f_{i}}E_{m}^{2}>\frac{1-\lambda_{i}}{\sigma_{RC}(p_{C}\;\text{ln}\;(E_{m})-1)}$. Then, the proposition 7 is as follow:

**Propostion 7** The regulatory penalty rate under the single constraint policies is higher than under the coexisting incentive and constraint policy, which requires the CSRC to impose higher penalties to guarantee fair rating and certification information from GECAs and CRAs. However, higher penalties result in higher regulatory costs. When only strict regulatory constraints are considered and no financial subsidies are provided by local governments, *E*_*m*_ is a crucial factor for fair rating results. At this time, $E_{m}\geq {\left(\frac{1}{p_{i}}f_{i}\right)}^{\frac{1}{2}}$. The proof 7 is in S1 Appendix.

### 3.3 Under the incentive policies

#### Scenario 9:Fair strategies

When both two agencies choose fair strategies, the expected profits of two agencies are as follow:

$$\begin{array}{c} \begin{array}{c} R_{i}=[1+r_{m_{1}}(p_{i}E_{m}-1)]\frac{1+\lambda_{i}}{2}f_{i}-\frac{\lambda_{i}p_{i}}{2}E_{m}^{2} \end{array} \end{array}$$ (18)

**Propostion 8** Under the incentive mechanism, with condition $r_{m_{1}}\in [\frac{2\lambda_{i}E_{m}}{f_{i}(1+\lambda_{i})},1]$ satisfied, GECAs and CRAs will choose the fair strategies of rating and certification with a high probability.

The proof 8 is in S1 Appendix.

#### Scenario 12:“Greenwashing” and “Inflated” rating

In this scenario, the expected profit of two agencies is as follow:

$$\begin{array}{c} \begin{array}{c} R_{i}=(1+p_{i}E_{m})(1-\lambda_{i})(1+r_{m1}u_{m})f_{i}-\frac{\lambda_{i}p_{i}}{2}E_{m}^{2}-\sigma_{RC}(1+r_{m1}u_{m})f_{i} \end{array} \end{array}$$ (19)

**Proposition 9** Under the incentive mechanism, GECAs and CRAs are more likely to issue “greenwashing” certification or “inflated” rating results when $r_{m_{1}}\in [\frac{\lambda_{i}E_{m}-(1-\lambda_{i})f_{i}}{(1-\lambda_{i})f_{i}u_{m}},\frac{2\lambda_{i}E_{m}}{f_{i}(1+\lambda_{i})})$ and $E_{m}\in [\frac{\sigma_{RC}-(1-\lambda_{i})}{(1-\lambda_{i})p_{i}},\frac{1}{p_{i}})$.

The proof 9 is in S1 Appendix.

According to the values we obtained from scenario 9 and 12, we can get lemma 5:

**Lemma 5** Considering only financial incentives and ignoring strict regulatory constraints, the conditions for fair strategy choice by GECAs and CRAs is $E_{m}\in [\frac{1}{p_{i}},\frac{(1-\lambda_{i}^{2})f_{i}}{\lambda_{i}(1+\lambda_{i}-2(1-\lambda_{i})u_{m})}]$.

The proof of Lemma 5 is in S1 Appendix.

## 4. Numerical analysis and simulation

### 4.1 Analysis of policy influencing factors

The above discussion through the theoretical exploration of the strategic choices and expected utilities of green certification agencies and credit rating agencies in 9 different scenarios in the Stackelberg game. The paper horizontally compares the strategic choices of rating agencies under the coexisting incentive and constraint policy, showing the effectiveness of different incentive and constraint policies. Vertically, the paper compares the key influencing factors of different incentive and constraint policies, providing a reference for regulatory agencies to choose appropriate incentive and constraint policies in different situations.

By comparing the impact factors of three different incentive and constraint policies: coexisting incentive and constraint policy, single strict regulation constraint policy and single financial incentives policy, it can be seen that the strategic choices of green certification agencies and credit rating agencies are mainly influenced by $r_{m_{1}}$, $r_{m_{2}}$, and *E*_*m*_. The environmental benefit factor and the local government financial subsidies $r_{m_{1}}$ under the single financial incentive policy, and the regulatory penalty rate $r_{m_{2}}$ under the strict regulation constraint policy are closely related.

Comparing the financial incentives in the coexisting incentive constraint policy and the single financial incentive policies, it can be seen that when the green certification agencies and credit rating agencies choose a fair rating certification strategy, the optimal incentive subsidy coefficient $r_{m_{1}}$ of the local government is the same. By comparing $r_{m_{1}}$ of the rating agencies choosing unfair rating and certification strategies under the single financial incentive policies and the coexisting policy, it is found that the local government under the single financial incentive policy needs to provide more financial incentives than the local government under the incentive and constraint policy to encourage the green certification agencies and credit rating agencies to provide fair green bond issuance rating and certification information. That is, the financial incentive cost of the local government under the single financial incentive policy is greater than that under the coexisting incentive and constraint mechanism. Therefore, the optimal strategy for the local government is to cooperate with the regulatory agency and provide both financial incentives and regulatory constraints to improve the incentive efficiency and reduce the incentive cost.

For the proof of this analysis, please see S1 Appendix.

Similarly, the comparison between the regulatory penalty rate $r_{m_{2}}^{*}$ under the co-exist of incentives and constraints and under the single strict regulatory constraint is made. The size of $r_{m_{2}}^{*}$ under different policies cannot be determined solely by numerical comparison. Analysis shows that the co-exist of incentives and constraints and the single strict regulatory constraint have different conditions of application. Under the condition of meeting other criteria, both policy measures can effectively promote green certification agencies and credit rating agencies to provide effective evaluation and rating information during the green bond issuance process.

For the proof of this analysis, please see S1 Appendix.

It can be seen that under the single financial incentives policy, the financial incentives strategy of the local government towards green bond issuers can effectively guide the GECAs and CRAs to issue accurate evaluations and ratings when $p_{i}\in (\frac{1}{f_{i}},1)$, compared to the single strict regulatory constraints policy.

For the proof of this analysis, please see S1 Appendix.

### 4.2 Simulation

[30]used data simulation to verify the influence of dual reputation incentives on the quality of major construction projects, and [11] used data simulation to analyze the regulatory effect of the collusion incentive and restraint mechanism of rating agencies. Applying the simulation methodologies from [11, 30], we utilize the simulations with MATLAB to examine the Lemma 1 to Lemma 4 in Section 3. Our primary objective is to illustrate the regulatory effect of three incentive and constraint policies. Therefore, we have fine-tuned these parameters to dynamically assess the impact of key determinants on the strategic choices of CRAs and GECAs.

The design of our simulation parameters’ initial values adheres to two fundamental principles: strict compliance with the parameter ranges presented in Model Assumptions, and determination of relevant parameters by referring to the concrete context of China’s green bond market along with pertinent academic research literature. In light of the empirical conditions of China’s rating industry and the simulation analysis of bond rating regulatory effects conducted by [11, 27], we have ascertained the initial values for the subsequent parameters: *f*_*i*_=10, *u*_*m*_=5, and λ_*i*_=0.25. The studies by [31, 32] that explore the weighting of environmental benefit indicators in the rating and information disclosure models inform our initial value assignment. Therefore, under both incentive and constraint policies, we set the initial value of *p*_*i*_=0.5; whereas under the single incentive or constraint policies, we assign *p*_*i*_=0.25. Previous analyses by [25, 33] propose a positive correlation between the regulator’s discount factor size and the intensity of regulatory penalties. Based on this premise, we assign the regulator’s discount factor initial value as follows: under both incentive and constraint policies or single regulation policies, *σ*_*RC*_=0.9; under the singular incentive policies, *σ*_*RC*_=0.1.

**Fig 5:**
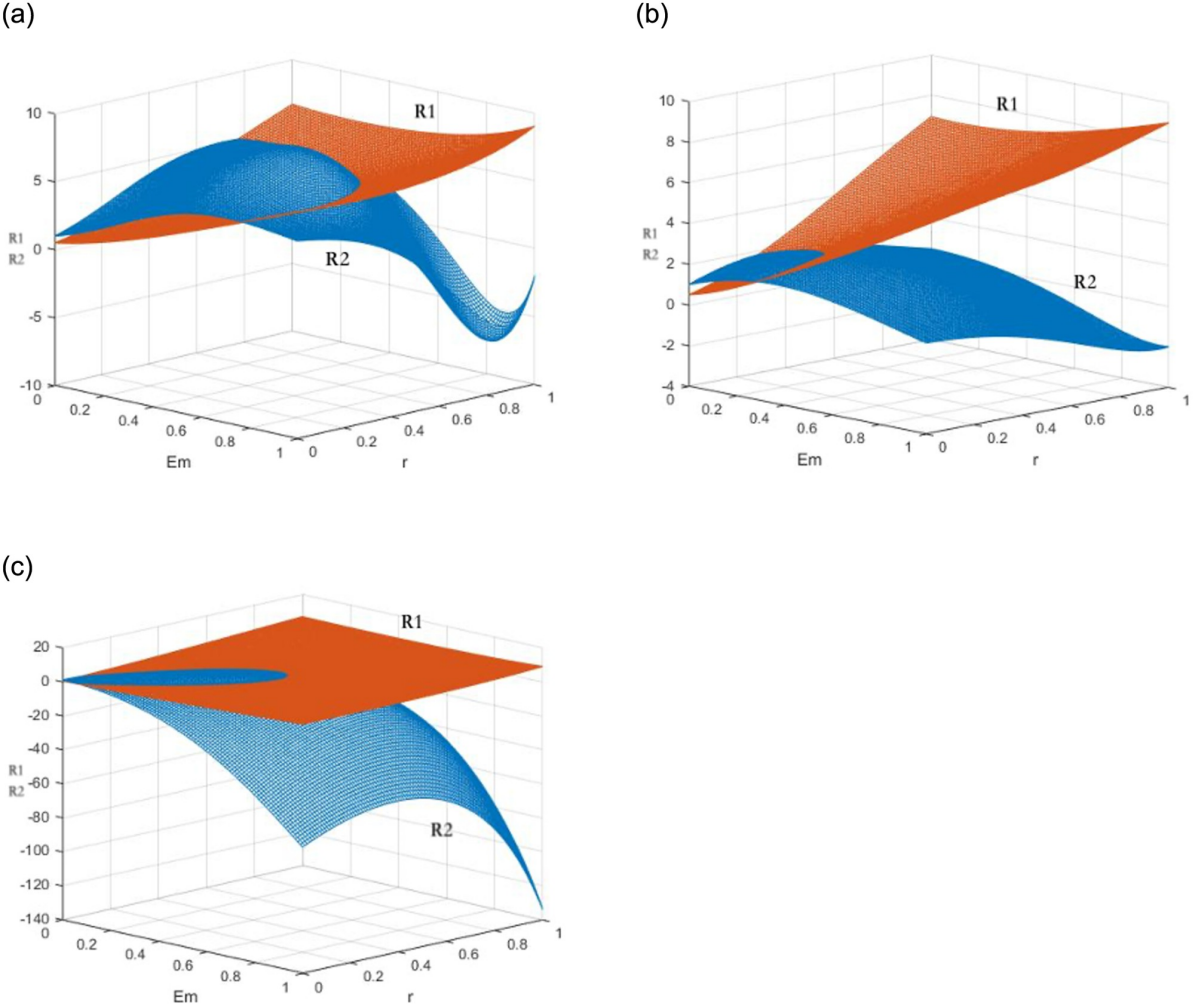
The expected returns of GECA and CRA under three incentive and constraint policies. (a) scenario 1 and 4, (b) scenario 5 and 8, (c) scenario 9 and 12.

For the results, The X, Y, and Z axes represent CRAs’ and GECAs’ expected profits, environmental benefits *E*_*m*_, and the policy coefficients *r*_*m*_ respectively. The expected profits of two agencies with fair strategy and unfair one is reported in Fig 5. Fig 5a shows the expected profits *R*_1_ of GECAs and CRAs under the fair certification and rating strategy and the expected profits *R*_2_ of colluding to issue “greenwashing” certification and “inflated” rating under the coexisting policy. Indeed, with the increase of *r*_*m*_ and *E*_*m*_, the surface *R*_1_ rises slowly, while the surface *R*_2_ decreases rapidly. The two briefly intersect when $r_{m_{1}}=\frac{2{\lambda_{i}E}_{m}}{f_{i}\left(1+\lambda_{i}\right)}$. Hereafter, the fair strategy profits *R*_1_ surpasses the unfair one *R*_2_. The same happens in strict regulation mechanism (see Fig 5b) and incentives mechanism (see Fig 5c).

The results of the study show that the impact of financial incentives and regulatory constraints on the quality of green bond ratings and certifications varies greatly. The influence of environmental benefits is more significant than that of financial incentives. The profit surfaces change more gradually under the coexistence of incentives and regulatory penalties, while they change more quickly under the strict constraint mechanism. The expected utility growth of rating agencies is the highest under the incentive mechanism, while the strict regulation mechanism is more effective in achieving fair certification and rating information.

Financial incentives can effectively increase the amount of green bonds issuance in the early stage of the green bond market, but the cost for the government is high. The local government should carefully consider the cost of incentives to avoid the risk of “greenwashing” and “inflated” ratings. As the green bond market develops, the single constraint mechanism can prevent such practices. The goal of the regulator is to transition from an incentive-constraint mechanism to single constraint mechanism. The results of this simulation support the conclusions of Lemma 1 and 2.

**Fig 6:**
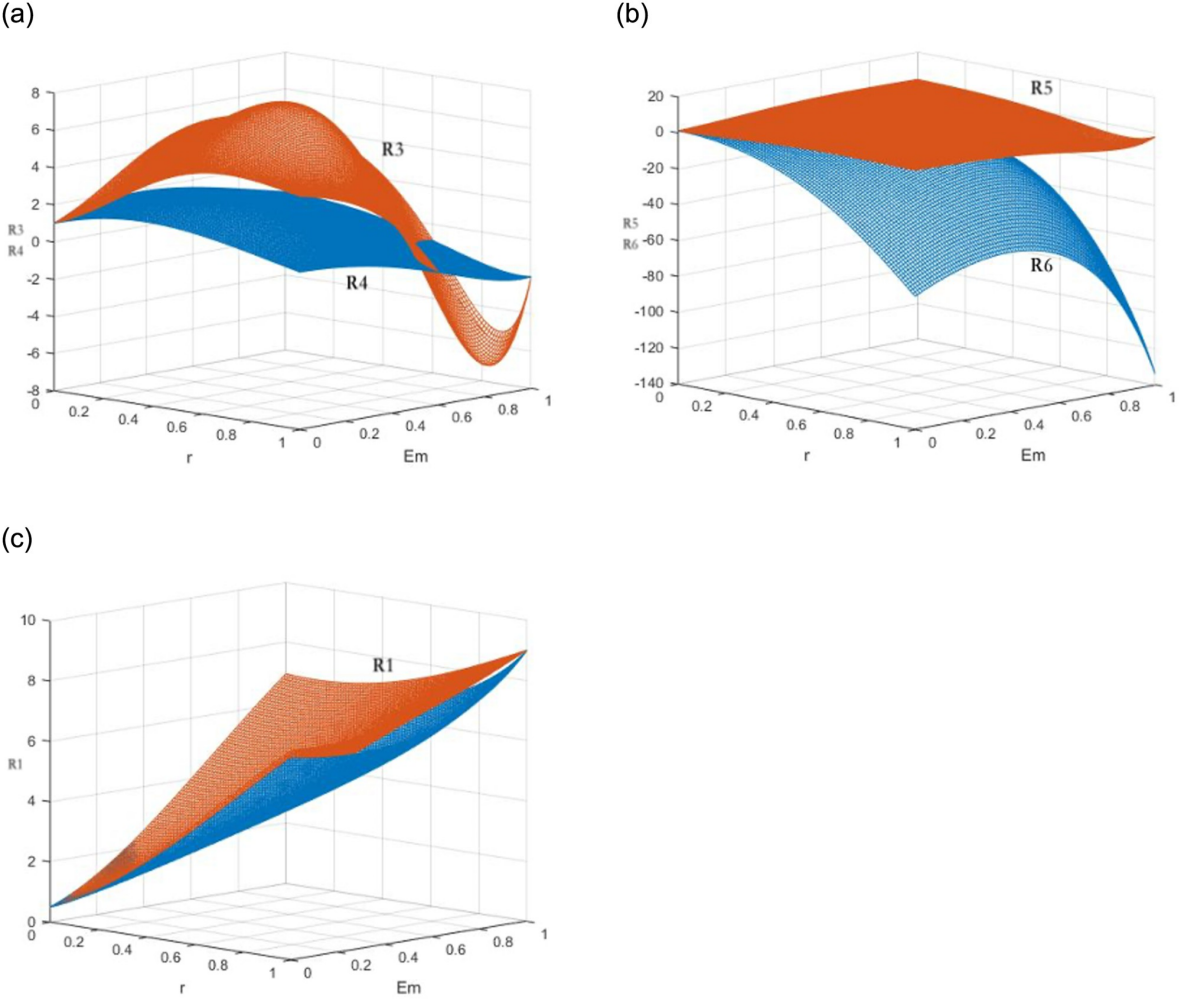
Expected returns of the same strategy of GECA under three incentive and constraint policies. (a) scenario 4 and 8, (b) scenario 4 and 12, (c) scenario 5 and 9.

The expected profits of the two agencies under “greenwashing” and “inflated” rating strategies are reported in Fig 6a and 6b, while the expected profits under a fair strategy are reported in Fig 6c. The results show that as the value of *E*_*m*_ increases, the two institutions are more likely to provide fair ratings and reduce the risk of punishment. At the same time, the local government does not need to provide a larger financial incentive to encourage fair strategies.

The CSRC and local government consider the environment effects and low-carbon subsidies when choosing regulation mechanisms. Compared to the strict regulation mechanism, the expected utility of the unfair strategy under the incentive-constraint mechanism changes more widely. It is shown from Fig 6b that the influence from local government’s financial incentives is less than the regulatory penalties by the CSRC for unfair rating and certification. The proportion of environmental effects in the rating system can effectively motivate GECAs and CRAs to choose impartial ratings. Local governments aim to increase the weight of environmental effects in the rating and certification system through financial incentives and low-carbon subsidies. The expected utility of fair strategy under two mechanisms is similar in Fig 6c. Regulators need to promote accurate rating and certification information by controlling the expected utility of institutions under unfair ratings and improving their utility under fair ratings, to prevent “greenwashing” certification and “inflated” ratings. The results of this simulation support the conclusions of Lemma 3 and 4.

## 5. Empirical analysis

In section 3 and 4, we investigate the impact of three different policy approaches: single incentive policies, single constraint policies, and a coexisting incentive-constraint policies on the quality of green bond rating and evaluation information. We explore this topic using Stackelberg game model and numerical simulations. At the theoretical level, we provide an analysis and discussion of the effects of these three policies. Furthermore, we employ an empirical analysis in this section by selecting data from China’s green bond market from 2016 to 2021, enabling us to examine the regulatory effects of each policy individually. This empirical analysis provides a stronger foundation for the research findings of this paper.

This section considers the number of local government financial incentives policies issued in 31 provinces and cities from 2016 to 2021 as single financial incentives measure, and the number of green bond regulatory constraint policies issued by the CSRC from 2016 to 2021 as single regulatory constraints measure. Since the *Green System Guidelines* involves both financial incentives and regulatory constraints, this chapter considers *Green System Guidelines* as a coexistence of incentives and constraints. Based on the game equilibrium analysis conclusion and numerical study, the following empirical hypotheses are proposed:

H1: Single financial incentive policies can effectively prompt green bond evaluation and certification agencies to upgrade their certification conclusions.
H2: Single regulatory constraint policies can prompt green bond credit rating agencies to downgrade their rating results.
H3: The coexisting incentive-constraint policies can prompt rating and certification agencies to downgrade their rating and certification results, effectively reducing the “greenwashing” and “inflated” phenomena of green bonds.

### 5.1 Data processing

To examine the impact of single financial incentive policies, single regulatory constraint policies, and coexisting incentive-constraint policies on the green bond rating and certification results, we selected 327 green bonds issued by green companies in China from 2016 to 2021 as sample for empirical analysis. After excluding samples that were not evaluated or rated for green or had missing control variables, 143 green bonds remained. The green certification of the green bonds were manually collected from the bond prospectus, while the annual green bond incentives in each province and city were sourced from the Syntao Green Finance Policy Database, and the regulatory constraint policies released by the CSRC each year were obtained from the CSRC’s official website. The green bond development indices of each province and city in China were calculated as the proportion of the total green bond issuance in each province and city among all bond issuances. The related data was sourced from the Statistical Yearbooks of each province and city in China and the China Financial Yearbook. The related financial information of the green bonds were sourced from the Wind database.

The explanatory variables include single financial incentive policies (*Incentive*_*s*_), single regulatory constraint policies (*Regulation*_*s*_), and coexisting incentives-constraints (*Incen* − *Cons*).

The control variables include bond term (*Term*), bond issuance scale (*Size*), green bond fundraising direction (*ESGF*), asset-liability ratio (*Leverage*), total asset return rate (*ROA*), operating profit rate (*CFO*), net asset return rate (*ROE*), corporate asset size (*Size*_*b*_), and green bond development index (*Index*_*green*_). The green bond development index is valued from high to low, with the best green bond development performance being 31 and the second-best being 30. In order of grades, the lowest green bond development performance of 31 provinces and cities nationwide is 1. The specific explanations of the variables is shown in Table 1.

**Table 1:** The definition of variables.

**Dependent variables**		
Variables	Symbol	Definition
Green Bond Credit Rating	*Rating*	Transformed to numerical values from low to high, i.e., AAA-rated green bonds are 3, AA+ rated are 2, and AA-rated are 1
Green Bond Evaluation Conclusion	*Evaluation*	Having evaluation certification conclusion is assigned as 1, otherwise 0. Based on the green level given by each green evaluation agency, values are assigned from high to low, with deep green G1, Ge-1, and GB1 as 3, green G2, Ge-2, and GB2 as 2, and lighter green G3, Ge-3, and GB3 as 1
**Independent variables**		
Variables	Symbol	Definition
Single Fiscal Incentive Policy	*Incentive*_*s*_	The number of incentive policies for green bond issuance in each province in that year
Single Regulatory Constraint Policy	*Regulation*_*s*_	The number of regulatory constraint policies issued by the CSRC for green bonds in that year
Incentive Constraint Policy	*Incen* − *Cons*	Set as a dummy variable. The value is 1 after the issuance of the “Green System Guidance” on February 2, 2021, otherwise 0
**Controlled variables**		
Variables	Symbol	Definition
Issuance Term	*Term*	Numerical value of bond issuance term, taking the natural logarithm
Issuance Size	*Size*	Bond issuance amount (billion), taking the natural logarithm
Green Bond Fundraising Investment	*ESGF*	Amount of funds raised for green projects (%)
Debt-to-Asset Ratio	*Leverage*	The percentage of total corporate liabilities to total corporate assets
Return on Total Assets	*ROA*	The ratio of net profit to total assets of the issuing company
Operating Profit Margin	*CFO*	Operating profit / operating revenue
Return on Equity	*ROE*	Net income divided by shareholder equity
Company Asset Size	*Size*_*b*_	Total assets of the issuing company, taking the natural logarithm
Provincial Green Bond Development Index	*Index*_*green*_	Provincial green bond issuance total amount / total amount of all bond issuances in the province, assigned values from high to low, with the highest development index as 31, and the lowest as 1

Stata was used to conduct descriptive statistics on the sample data, and the descriptive statistics of 12 variables before and after the issuance of the “Green System Guidelines” is shown in Table 2.

**Table 2:** Descriptive statistics of variables before and after the introduction of *Green System Guidance*.

	Before introduction				After introduction			
	mean	sd	min	max	mean	sd	min	max
*Rating*	2.255	1.163	0	3	1.88	1.333	0	3
*Evaluation*	1.02	0.316	0	3	1.152	0.592	0	3
*Index*_*green*_	0.385	0.124	0.147	0.564	0.353	0.153	0.014	0.603
*lnSize*	0.945	0.278	0	1.301	0.842	0.315	-0.301	1.699
*Term*	5.471	2.901	3	20	4.391	2.965	2	27
*ESGF*	84.216	15.112	65	100	81.07	14.571	50	100
*Leverage*	59.757	12.225	33.104	88.416	61.111	14.966	23.067	88.402
*ROA*	3.522	2.455	-0.67	9.351	2.489	2.787	-2.969	15.652
*CFO*	22.668	20.983	-12.724	112.851	9.892	47.994	-349.672	64.584
*ROE*	4.858	5.122	-4.043	24.558	4.223	5.609	-12.694	19.714
*lnSize*_*b*_	2.981	0.562	1.646	3.987	2.834	0.626	1.444	4.278

The *Green System Guidelines* was introduced on February 2, 2021, so the period from January 1, 2016 to February 1, 2021 was taken as the period before the introduction and the period from February 2, 2021 to December 31, 2021 was taken as the period after the introduction. As can be seen from the descriptive statistics, after the introduction, the average of bond credit ratings decreased from 2.255 to 1.88, and the standard deviation increased from 1.163 to 1.333. This indicates that the overall credit rating results have been lowered and the distribution of ratings has become more dispersed after the introduction. On the other hand, the average of green certification conclusions increased from 1.02 to 1.152, and the standard deviation also increased from 0.316 to 0.592. This indicates that the overall green certification conclusions have been raised after the introduction of *Green System Guidelines*.

Overall, after the issuance of the *Green System Guidelines*, the average credit rating decreased and became more dispersed, while the average green evaluation certification increased, but the distribution became more uneven. The average debt-to-asset ratio also increased and became more dispersed. Additionally, the average bond issuance size and bond issuance term both decreased and became more dispersed, the average funds raised for investment decreased slightly and became more concentrated, the average total asset yield, operating profit margin, and net asset yield all decreased and became more dispersed. The size of the enterprise assets as a whole decreased and became more dispersed. The changes in these variables may be related to the policy orientation of the *Green System Guidelines* and reflect the policy’s impact on the credit rating, green certification, bond issuance size, term, fund allocation, debt-to-asset ratio, and profitability of green bonds and their issuers.

### 5.2 The ordered Logit model

The ordered Logit model is a widely used statistical model for analyzing relationships between ordinal categorical variables. Green bond credit ratings and certification conclusions are both multi-level ordinal qualitative indicators, with the rating and evaluation certification results divided into multiple levels that have distinct ordinal relationships [34] The ordered Logit model accommodates credit rating data characteristics and analyzes factors influencing credit ratings [35, 36]. Therefore, this study employs the ordered Logit model for empirical analysis.

Assuming there are *j* credit rating levels and green assessment certification levels, *y*_*i*_ represents the credit rating result of the *i*-th sample (*y*_*i*_ ∈ 1, 2, ⋯, *J*), and *x*_*i*1_, *x*_*i*2_, ⋯, *x*_*ik*_ denote the *k* independent variables of the *i*-the sample. The fundamental formula of the ordered Logit model is as follows:

$$\begin{array}{c} Pr\left(y_{i}\leq j|x_{i}\right)=\frac{\text{exp}\;(\alpha_{j}+\beta_{1}x_{i1}+\beta_{2}x_{i2}+\cdots +\beta_{k}x_{ik})}{1+\sum_{l=1}^{j-1}\;\text{exp}\;\left(\alpha_{l}+\beta_{1}x_{i1}+\beta_{2}x_{i2}+\cdots +\beta_{k}x_{ik}\right)} \end{array}$$ (20)

In this equation, *J* = 1, 2, ⋯, *j* − 1. *P*(*y*_*i*_ ≤ *J*|*x*_*i*_) denotes the probability that the credit rating result or green evaluation certification conclusion of the *i*-th sample is less than or equal to *j* under the given independent variable *x*_*i*_; *α*_*j*_ represents the intercept of the *j*-th rating and evaluation level; and *β*_1_, *β*_2_, ⋯, *β*_*k*_ signify the coefficients of the corresponding independent variables.

This paper initially examines the influence of single incentive or constraint policies on credit rating and green evaluation information, essentially evaluating if the rating or evaluation information reflects policy impacts. This model is shown in Eqs (21) and (22):

$$\begin{array}{c} \begin{array}{c} Rating_{i}=\alpha_{0}+\beta_{1}Incentive_{s_{i}}\left(Regulation_{s_{i}}\right)+\beta_{3}Term_{i}+\beta_{4}Size_{i}+\beta_{5}ESGF_{i}+\beta_{6}\\ Leverage_{i}+\beta_{7}ROA_{i}+\beta_{8}CFO_{i}+\beta_{9}ROE_{i}+\beta_{10}Size_{b_{i}}+\beta_{11}Index_{green_{i}}+\epsilon_{i} \end{array} \end{array}$$ (21)

$$\begin{array}{c} \begin{array}{c} Evaluation_{i}=\alpha_{0}+\beta_{1}Incentive_{s_{i}}\left(Regulation_{s_{i}}\right)+\beta_{3}Term_{i}+\beta_{4}Size_{i}+\beta_{5}ESGF_{i}+\\ \beta_{6}Leverage_{i}+\beta_{7}ROA_{i}+\beta_{8}CFO_{i}+\beta_{9}ROE_{i}+\beta_{10}Size_{b_{i}}+\beta_{11}Index_{green_{i}}+\epsilon_{i} \end{array} \end{array}$$ (22)

Among them, *Rating*_*i*_ and *Evaluation*_*i*_ represent the rating and evaluation conclusion of the i-th green bond. These two dependent variables are sequential discrete variables, for which definitions are provided in Table 1. The terms $Incentive_{s_{i}}$ and $Regulation_{s_{i}}$ represent, respectively, the annual count of incentive policies for green bond issuance in each province, and the annual count of regulatory constraint policies issued by the CSRC for green bonds. Drawing on the studies of [11, 34, 37], we categorize the factors influencing bond rating and evaluation into two subsets: issuer variables and debt variables. The issuer variables encompass *Index*_*green*_, *Size*_*b*_, along with financial variables *ESGF*, *Leverage*, *ROA*, *ROE*, and *CFO*. Debt variables include *Term* and *Size*. Detailed descriptions of these control variables are provided in Table 1.

Further, we also discuss the scenario where incentives and constraints coexist. Taking into account the authority and influence of the policy issuer, we have selected the *Green System Guidelines* as a representative of incentive-constraint policies and treated it as a dummy variable. This variable takes on a value of 1 for green bond issues post the issuance date of February 2, 2021, and 0 otherwise. The following models are utilized to examine the impact of the *Green System Guidelines* on green bond credit rating and certification conclusions pre and post its promulgation:

$$\begin{array}{c} \begin{array}{c} Rating_{i}=\alpha_{0}+\beta_{1}Incen-Cons+\beta_{3}Term_{i}+\beta_{4}Size_{i}+\beta_{5}ESGF_{i}+\beta_{6}\\ Leverage_{i}+\beta_{7}ROA_{i}+\beta_{8}CFO_{i}+\beta_{9}ROE_{i}+\beta_{10}Size_{b_{i}}+\beta_{11}Index_{green_{i}}+\epsilon_{i} \end{array} \end{array}$$ (23)

$$\begin{array}{c} \begin{array}{c} Evaluation_{i}=\alpha_{0}+\beta_{1}Incen-Cons+\beta_{3}Term_{i}+\beta_{4}Size_{i}+\beta_{5}ESGF_{i}+\beta_{6}\\ Leverage_{i}+\beta_{7}ROA_{i}+\beta_{8}CFO_{i}+\beta_{9}ROE_{i}+\beta_{10}Size_{b_{i}}+\beta_{11}Index_{green_{i}}+\epsilon_{i} \end{array} \end{array}$$ (24)

The selection of control variables in this model aligns with those used in Eqs (21) and (22). The error term *ϵ*_*i*_ is distributed independently of the individual and follows the Logistic cumulative distribution function [38].

### 5.3 Results

Table 3 presents the empirical results of the effects of local government incentives policies and CSRC regulatory constraints policies on the ratings and certifications of green bonds.

**Table 3:** The influence of rating and evaluation certification result under single financial incentive policies and single regulatory constraint policies.

Factors	*Rating*	*Evaluation*	*Rating*	*Evaluation*
	(1)	(2)	(3)	(4)
*Incentive*_*s*_	0.144*	0.160**		
	(1.95)	(2.03)		
*Incentive*_*c*_			-0.528**	0.097
			(-2.19)	(0.31)
*Index*_*green*_	0.715	-2.499	2.500	-2.046
	(0.42)	(-1.24)	(1.48)	(-1.07)
*lnSize*	2.183***	2.844***	2.204***	2.667***
	(3.26)	(2.77)	(3.27)	(2.62)
*Term*	0.013	-0.115*	0.016	-0.099
	(0.18)	(-1.67)	(0.22)	(-1.64)
*ESGF*	0.015	-0.045*	0.012	-0.041
	(1.12)	(-1.74)	(0.93)	(-1.63)
*Leverage*	0.053***	0.022	0.050***	0.023
	(2.79)	(0.71)	(2.98)	(0.75)
*ROA*	0.291**	-0.010	0.345**	0.003
	(2.13)	(-0.05)	(2.39)	(0.01)
*CFO*	0.003	0.002	0.002	0.005
	(0.43)	(0.32)	(0.28)	(0.78)
*ROE*	-0.054	0.024	-0.078	-0.008
	(-0.55)	(0.19)	(-0.82)	(-0.07)
*lnSize*_*b*_	1.357***	-1.286**	1.290***	-1.275**
	(2.82)	(-2.28)	(3.00)	(-2.40)
Constant *C*_1_	10.098***	-9.738***	11.456***	-8.933***
	(4.31)	(-3.10)	(5.00)	(-3.09)
Constant *C*_2_	10.355***	-2.458	11.720***	-1.844
	(4.42)	(-0.82)	(5.10)	(-0.66)
Constant *C*_3_	11.337***	-2.085	12.725***	-1.477
	(4.72)	(-0.71)	(5.34)	(-0.54)

Note: The values in the brackets are the z test statistics,

* represents the 10% level Significant,

** means significant at 5% level,

*** means significant at 1% level.

As shown in columns (1) and (2) of Table 3, the number of local government financial incentives is positively correlated with the credit ratings scores of green bonds at the 10% significance level and with the green evaluation certifications scores at the 5% significance level. This indicates that single financial incentives policies has a significant positive effect on the credit ratings and green evaluation certifications of green bonds. The bond issuance scale has a significant positive effect on both the credit ratings scores and the green evaluation certifications, meaning that companies with a larger bond issuance scale are more likely to receive higher credit ratings and green evaluation certifications. The size of the company’s assets has a significant positive effect on the credit ratings, but a significant negative effect on the green evaluation certifications. This supports the assumption of H1.

As shown in columns (3) and (4) of Table 3, the number of CSRC regulatory constraints policies has a negative correlation with the credit ratings results at the 5% significance level, but has no significant effect on the green evaluation certifications scores. This indicates that single regulatory constraints policies will lower the credit ratings results, but has no significant effect on the green evaluation certifications. Factors such as the size of the company’s green bond issuance, debt-to-asset ratio, and total asset return rate have a positive effect on the credit ratings, while the size of the company’s assets has a negative effect on the green evaluation certifications scores. This supports the assumption of H2.

Comparing the effects of single financial incentives policies and single regulatory constraints policies on the ratings and certifications of green bonds, as shown in Table 3, the probability of single financial incentives leading to an upward adjustment of the green evaluation certifications is 0.53581 ($\frac{e^{0.144}}{1+e^{0.144}}$), while the probability of single regulatory constraints leading to an upward adjustment is 0.37094 ($\frac{e^{-0.528}}{1+e^{-0.528}}$) times. This suggests that under the promotion of single financial incentives, the evaluation certification institutions may be more likely to adjust the green evaluation certifications upward, thereby obtaining financial incentives such as government subsidies and tax benefits. This empirical conclusion supports the assumption of H1. The credit ratings of green bonds have a significant asymmetrical effect under single financial incentives policies and single regulatory constraints policies, with the implementation of single financial incentives promoting the upward adjustment of the credit ratings of green bonds, while the implementation of single regulatory constraints promoting the downward adjustment of the credit ratings of green bonds. This reflects the impact of different policy environments on the quality of information on the credit ratings of green bonds. From a market perspective, single regulatory constraints policies will make credit rating institutions more cautious in their expectations of the credit risks of green bonds, resulting in a downward adjustment of the credit ratings results. This empirical conclusion supports the assumption of H2.

Table 4 presents the empirical results of the impact of *Green System Guidance* on the assessment of green bond ratings. The introduction has a significantly negative impact on the credit rating of green bonds, while its impact on the score of green assessment and certification is also negative but not significant. This implies that the coxisting incentive constraint policy has a significant impact on the downward adjustment of the credit rating of green bonds, while its impact on the downward adjustment of the conclusion of green certification is relatively weak.

**Table 4:** Impact results of the *Green System Guidance* on green bond rating assessment.

Variable	*Rating*	*Evaluation*
*Incen* − *Cons*	-1.050**	-0.441
	(-2.39)	(-0.81)
*Index*_*green*_	1.791	-2.046
	(1.22)	(-1.05)
*lnSize*	1.905***	2.650**
	(2.67)	(2.56)
*Term*	0.004	-0.100*
	(0.06)	(-1.72)
*ESGF*	0.018	-0.041*
	(1.39)	(-1.72)
*Leverage*	0.050***	0.025
	(2.77)	(0.81)
*ROA*	0.206	0.013
	(1.56)	(0.07)
*CFO*	0.004	0.006
	(0.53)	(0.98)
*ROE*	-0.047	-0.023
	(-0.46)	(-0.20)
*lnSize*_*b*_	1.299***	-1.299**
	(2.99)	(-2.43)
Constant *C*_1_	8.667***	-8.940***
	(4.23)	(-3.10)
Constant *C*_2_	8.927***	-1.799
	(4.37)	(-0.65)
Constant *C*_3_	9.931***	-1.433
	(4.72)	(-0.52)

Note: The values in the brackets are the z test statistics,

* represents the 10% level Significant,

** means significant at 5% level,

*** means significant at 1% level.

The size of bond issuance exerts a notably positive impact on credit ratings and green assessment certification conclusions. This may suggest that bond issuers with larger issuance scales typically possess higher credit ratings and green certifications. The allocation of funds towards bond issuance duration and corporate asset scale has a significantly negative influence on the green bond assessment certification conclusions. Financial indicators, such as the asset-liability ratio and the return on total assets, demonstrate a positive effect on the credit ratings of green bonds.

Comparing the data in Tables 3 and 4, it is evident that single financial incentive policies significantly increases the credit rating and green assessment conclusions of green bonds. Single regulatory constraint policies has a significant negative impact on credit rating scores, but no significant impact on green assessment certification scores. When incentive and constraint policies coexist, there is a significant negative effect on credit rating scores, while no significant impact on green assessment certification scores. This implies that the promulgation of the “Green System Guidance” may result in a downgrade of green bond credit ratings, but the impact on the downgrade of green assessment certification conclusions is limited. Comparing the effects of single incentive and constraint policies and their coexistence on green certification conclusions, it is clear that the introduction of the *Green System Guidance* still has a weak positive influence on the downgrade of green assessment certification conclusions, while the single incentive or constraint policies have no impact on the downgrade of green assessment certification conclusions. This suggests that, compared to single incentive or constraint policies, the coexisting incentive and constraint policies can prompt rating and certification agencies to approach the evaluation of green bond rating and certification information more cautiously. This empirical result is consistent with the theoretical analysis conclusion obtained in the above section.

### 5.4 Robustness test

To confirm the robustness of the empirical results, this study follows the research of [37] and employs the ordinary least squares (OLS) estimation method to perform a robustness test on Eqs (21) to (24).

The robustness test result presents the results of the impact of incentive and constraint policies on green bond rating and certification results based on the OLS estimation method. A comparative analysis of Tables 3–5. By comparing columns (1) and (2) of Table 3 with columns (1) and (2) of Table 5, it can be observed that the positive significance of local government green bond incentives remains at the 5% significance level. This result indicates a significant positive impact of policy incentives on rating and certification. Comparing columns (3) and (4) of Table 3 with columns (3) and (4) of Table 5, it can be seen that the implementation of single regulatory policies has a significant influence on the downgrading of green bond credit ratings.

**Table 5:** Robustness test.

	*Rating*	*Evaluation*	*Rating*	*Evaluation*	*Rating*	*Evaluation*
Variables	(1)	(2)	(3)	(4)	(5)	(6)
*Incentive*_*s*_	0.053*	0.027**				
	(1.76)	(2.00)				
*Regulation*_*s*_			-0.225**	0.10		
			(-2.20)	(0.31)		
*Incen* − *Cons*					-0.508***	-0.05
					(-2.77)	(-0.52)
*Index*_*green*_	0.44	(0.31)	1.067	-2.046	0.84	-0.33
	(0.54)	(-1.02)	-1.52	(-1.07)	(1.26)	(-1.04)
*lnSize*	1.111***	0.394**	1.133***	2.667***	0.972***	0.331**
	(3.56)	(2.32)	-3.62	-2.62	(3.10)	(2.35)
*Term*	0.00	(0.02)	-0.004	-0.099	0.00	-0.018*
	(0.01)	(-1.11)	(-0.14)	(-1.64)	(0.06)	(-1.93)
*ESGF*	0.01	(0.29)	0.008	-0.041	0.01	0.00
	(1.23)	(-1.21)	-1.29	(-1.63)	(1.49)	(-1.19)
*Leverage*	0.027***	0.11	0.026***	0.023	0.027***	0.00
	(3.92)	(0.55)	-3.92	-0.75	(3.79)	(0.69)
*ROA*	0.103*	(0.01)	0.127**	0.003	0.090*	0.00
	(1.93)	(-0.34)	-2.21	-0.01	(1.70)	(-0.13)
*CFO*	0.01	0.00	0.001	0.005	0.00	0.00
	(0.52)	(0.32)	-0.38	-0.78	(0.87)	(1.16)
*ROE*	(0.01)	0.01	-0.023	-0.008	(0.02)	0.00
	(-0.39)	(0.53)	(-0.74)	(-0.07)	(-0.65)	(-0.00)
*lnSize*_*b*_	0.484***	-0.215**	0.466***	-1.275**	0.475***	-0.190**
	(2.89)	(-2.40)	-2.8	(-2.40)	(2.92)	(-2.41)
Constant	-3.246***	2.312*	-3.975***		-2.743***	1.700***
	(-3.96)	(1.66)	(-4.47)		(-3.24)	(3.73)
Constant cut1				-8.933***		
				(-3.09)		
Constant cut2				-1.844		
				(-0.66)		
Constant cut3				-1.477		
				(-0.54)		
R-squared	0.38	0.11	0.383		0.398	0.084

Note: The values in brackets are t-test statistics,

* means significant at 10% level,

** means significant at 5% level,

*** means significant at 1% level.

However, after the implementation of the *Green System Guidance*, the regression coefficients for green bond credit ratings remain significantly negative, while the impact on green bond evaluation certification results is not significant, but the coefficients are still negative.

Although there are differences in significance, these conclusions are consistent with the corresponding empirical results in Tables 3 and 4, indicating that the results of H1 to H3 are robust.

## 6. Conclusions and research prospect

Based on the policy environment of China’s green bond issuance market, this paper classifies incentive and constraint policies implemented by governments and regulatory authorities into three categories: single financial incentive policies, single regulatory restraint policies, and combined incentive and constraint policies. Within the integrated rating and certification regulation mechanism, we construct a two-stage incentive-constraint Stackelberg game model to explore the effects of these diverse policies on the quality of rating and certification during the green bond issuance process. Our approach integrates game analysis, simulation, and empirical analysis to offer a comprehensive understanding of how these policies impact rating and certification outcomes.

Several managerial insights can be drawn with the analysis of this study:(1) For both rating and certification agencies, the influence of local government incentives is substantially less significant than the regulatory penalties imposed by the CSRC. In the stable development phase of the green bond market, the most effective strategy to enhance the quality of green bond rating and certification information is through a single constraint mechanism, followed by a combined incentive-restraint mechanism, with the single incentive mechanism being the least effective. (2) The financial incentive costs for local governments under the single incentive mechanism are higher than those under the incentive-restraint mechanism. In the early stages of the green bond market, when financial incentives are necessary, the optimal strategy for local governments involves collaborating with the CSRC to provide both financial incentives and regulatory constraints on green bonds. This approach improves incentive efficiency while reducing costs. (3) While financial incentives alone cannot exert a strong direct impact on impartial ratings, increasing the proportion of environmental effects in the rating system can effectively encourage GECAs and CRAs to adopt impartial ratings.

Local governments should concentrate on identifying methods to increase the weight of environmental effects in the rating and certification system through financial incentives and low-carbon subsidies. And regulators should focus on striking a balance between fines, inspection frequency, and regulatory costs, ensuring that the incentive environment for green bonds does not lead to a relaxation of regulation. By analyzing the green bond issuance market, social funds can be channeled towards investments in projects with substantial positive environmental benefits, utilizing financial means to support the efficient allocation of environmental resources.

## Supporting information

S1 Appendix This is the supplement of the research. (DOCX)
