# Supplementary material for: Effectivenes of incentive constraint policies in enhancing green bond credit rating and certification: A theoretical and empirical study
Source: PLoS One. 2023 Nov 16;18(11):e0289750. doi: 10.1371/journal.pone.0289750 (PMC10653487; doi:10.1371/journal.pone.0289750)
Supplement: S1 Appendix — (DOCX) [file pone.0289750.s001.docx]

**Proof 1 of proposition 1:**

In this scenario, we calculate the optimal response of two agencies:

|  | $\frac{\partial R_{i}}{\partial r_{m_{1}}}=\frac{1}{2}(p_{i}E_{m}-1)(1+\lambda_{i})f_{i}$ |  |
| --- | --- | --- |
|  | $\frac{\partial R_{i}}{\partial E_{m}}=\frac{1}{2}p_{i}(-2\lambda_{i}E_{m}+r_{m_{1}}f_{i}(1+\lambda_{i}))$ |  |

Then the value of $r_{m_{1}}^{*},E_{m}^{*}$ can be obtained by the above three Equations:

|  | $r_{m_{1}}^{*}=\frac{2{\lambda_{i}E}_{m}}{f_{i}(1+\lambda_{i})}$ |  |
| --- | --- | --- |
|  | $E_{m}^{*}=\frac{1}{p_{i}}$ |  |

**Proof 2 of proposition 2:**

The optimal responses are as follows:

| $\frac{\partial R_{geca}}{\partial r_{m_{1}}}=\frac{1}{2}f_{i}(p_{G}E_{m}-1)(1+\lambda_{i})\lambda_{i}$ |  |
| --- | --- |
| $\frac{\partial R_{cra}}{\partial r_{m_{1}}}=(1-\lambda_{i}){u_{m}f}_{i}+{\sigma_{RC}r}_{m_{2}}(p_{C}ln\left( E_{m} \right)-1)u_{m}f_{i}$ |  |
| $\frac{\partial R_{cra}}{\partial r_{m_{2}}}=\sigma_{RC}(p_{C}ln[E_{m}]-1)(1+u_{m}r_{m_{1}})f_{i}$ |  |

Then, $r_{m_{1}}^{*}(geca)$、$r_{m_{1}}^{*}(cra)$ and $r_{m_{2}}^{*}(cra)$ can be obtained by the above three Equations:

| $r_{m_{1}}^{*}(geca)=\frac{2{\lambda_{i}E}_{m}}{f_{i}(1+\lambda_{i})}$ |  |
| --- | --- |
| $r_{m_{1}}^{*}(cra)=-\frac{1}{u_{m}}$ |  |
| $r_{m_{2}}^{*}(cra)=\frac{1{-\lambda}_{i}}{\sigma_{RC}(p_{C}ln\left( E_{m} \right)-1)}$ |  |

**Proof 3 of proposition 3:**

The proof process for this scenario is similar to scenario 2. The optimal response are as follows:

| $\frac{\partial R_{geca}}{\partial r_{m_{1}}}=(1-\lambda_{i}){u_{m}f}_{i}+{\sigma_{RC}r}_{m_{2}}(p_{G}ln\left( E_{m} \right)-1)u_{m}f_{i}$ |  |
| --- | --- |
| $\frac{\partial R_{geca}}{\partial r_{m_{2}}}=\sigma_{RC}(p_{G}ln[E_{m}]-1)(1+u_{m}r_{m_{1}})f_{i}$ |  |
| $\frac{\partial R_{cra}}{\partial r_{m_{1}}}=\frac{1+\lambda_{i}}{2}(p_{i}E_{m}-1)f_{i}$ |  |

Therefore, the values can be obtained by the above three Equations:

| $r_{m_{1}}^{*}(geca)=-\frac{1}{u_{m}}$ |  |
| --- | --- |
| $r_{m_{2}}^{*}(geca)=\frac{1{-\lambda}_{i}}{\sigma_{RC}(p_{G}ln\left( E_{m} \right)-1)}$ |  |
| $r_{m_{1}}^{*}(cra)=\frac{2{\lambda_{i}E}_{m}}{f_{i}(1+\lambda_{i})}$ |  |

**Proof 4 of proposition 4:**

In this scenario, the optimal response of $R_{i}$ are as follows:

|  | $\frac{\partial R_{i}}{\partial r_{m_{1}}}=(1-\lambda_{i}){u_{m}f}_{i}-{\sigma_{RC}r}_{m_{2}}(1-p_{i}ln\left( E_{m} \right))u_{m}f_{i}$ |  |
| --- | --- | --- |
|  | $\frac{\partial R_{i}}{\partial r_{m_{2}}}=\sigma_{RC}(p_{i}ln\left( E_{m} \right)-1)(1+u_{m}r_{m_{1}})f_{i}$ |  |
|  | $\frac{\partial R_{i}}{\partial E_{m}}=\frac{{\sigma_{RC}r}_{m_{2}}(1+u_{m}r_{m_{1}}){p_{i}f}_{i}}{E_{m}}-p_{i}\lambda_{i}E_{m}$ |  |

Then, these values $r_{m_{1}}^{*}$、$r_{m_{2}}^{*}$ and $E_{m}^{*}$can be obtained by the above three Equations:

| $r_{m_{1}}^{*}=-\frac{1}{u_{m}}$ |  |
| --- | --- |
| $r_{m_{2}}^{*}=\frac{1-\lambda_{i}}{\sigma_{RC}(p_{i}ln\left( E_{m} \right)-1)}$ |  |
| $E_{m}^{*}=\left( \frac{{\sigma_{RC}r}_{m_{2}}(1+u_{m}r_{m_{1}})f_{i}}{\lambda_{i}} \right)^{\frac{1}{2}}$ |  |

**Proof 5 of proposition 5:**

In this scenario, we calculate the optimal response of two agencies:

|  | $\frac{\partial R_{i}}{\partial\lambda_{i}}=\frac{1}{2}f_{i}-\frac{1}{2}p_{i}E_{m}^{2}$ |  |
| --- | --- | --- |
|  | $\frac{\partial R_{i}}{\partial E_{m}}=-p_{i}\lambda_{i}E_{m}$ |  |

Then, the value of $E_{m}^{*}$ can be obtained by the above two Equations:

| $E_{m}^{*}=\left( \frac{1}{p_{i}}f_{i} \right)^{\frac{1}{2}}$ |  |
| --- | --- |

**Proof 6 of proposition 6:**

The optimal response of is as follows:

|  | $\frac{\partial R_{geca}}{\partial E_{m}}=-p_{G}\lambda_{i}E_{m}$ |  |
| --- | --- | --- |
|  | $\frac{\partial R_{cra}}{\partial E_{m}}=\frac{{\sigma_{RC}r}_{m_{2}}}{ⅇ_{m}}p_{C}f_{i}-p_{C}\lambda_{i}E_{m}$ |  |
|  | $\frac{\partial R_{cra}}{\partial r_{m2}}=\sigma_{RC}(p_{C}ln\left( E_{m} \right)-1)f_{i}$ |  |

Therefore, we obtained these values:

|  | $E_{m}^{*}(geca)=\left( \frac{1}{p_{G}}f_{i} \right)^{\frac{1}{2}}$ |  |
| --- | --- | --- |
|  | $E_{m}^{*}(cra)=e^{\frac{1}{p_{C}}} , \frac{1}{p_{C}}\in\left( -\pi,\pi\right)$ |  |
|  | $r_{m_{2}}^{*}(cra)=\frac{\lambda_{i}}{\sigma_{RC}f_{i}}E_{m}^{2}$ |  |

**Proof 7 for Proposition 7:**

In this scenario, the expected profit of two agencies is as follow:

| $R_{i}=(1-\lambda_{i})f_{i}-\frac{\lambda_{i}p_{i}}{2}E_{m}^{2}-{\sigma_{RC}r}_{m_{2}}\left( 1-p_{i}ln\left( E_{m} \right) \right)f_{i}$ |  |
| --- | --- |

Then, the optimal response are as follows:

|  | $\frac{\partial R_{i}}{\partial E_{m}}=\frac{{\sigma_{RC}r}_{m_{2}}}{ⅇ_{m}}p_{i}f_{i}-p_{i}\lambda_{i}E_{m}$ |  |
| --- | --- | --- |
|  | $\frac{\partial R_{i}}{\partial r_{m2}}=\sigma_{RC}(p_{i}ln\left( E_{m} \right)-1)f_{i}$ |  |

Therefore, we obtained these values:

|  | $E_{m}^{*}=e^{\frac{1}{p_{C}}} , \frac{1}{p_{C}}\in\left( -\pi,\pi\right)$ |  |
| --- | --- | --- |
|  | $r_{m_{2}}^{*}=\frac{\lambda_{i}}{\sigma_{RC}f_{i}}E_{m}^{2}$ |  |

It can be seen from scenario 4 and scenario 8 that the value of regulatory penalty rate $\frac{\lambda_{i}}{\sigma_{RC}f_{i}}E_{m}^{2}>\frac{1{-\lambda}_{i}}{\sigma_{RC}(p_{C}ln\left( E_{m} \right)-1)}$ when the GECAs and CRAs choose unfair rating and certification.

**Proof 8 for Proposition 8:**

In these scenarios, we calculate the optimal response of two agencies:

|  | $\frac{\partial R_{i}}{\partial r_{m_{1}}}=\frac{1}{2}(p_{i}E_{m}-1)(1+\lambda_{i})f_{i}$ |  |
| --- | --- | --- |
|  | $\frac{\partial R_{i}}{\partial E_{m}}=\frac{1}{2}p_{i}(-2\lambda_{i}E_{m}+r_{m_{1}}p_{i}(1+\lambda_{i})f_{i})$ |  |

Therefore, we can obtain these values $r_{m_{1}}^{*}$ and $E_{m}^{*}$ by the above Equations:

|  | $r_{m_{1}}^{*}=\frac{2{\lambda_{i}E}_{m}}{f_{i}(1+\lambda_{i})}$ |  |
| --- | --- | --- |
|  | $E_{m}^{*}=\frac{1}{p_{i}}$ |  |

**Proof 9 for Proposition 9:**

In this scenario, we calculate the optimal response of two agencies:

|  | $\frac{\partial R_{i}}{\partial r_{m_{1}}}=-\sigma_{RC}{u_{m}f}_{i}+(1-\lambda_{i})(1+p_{i}E_{m})u_{m}f_{i}$ |  |
| --- | --- | --- |
|  | $\frac{\partial R_{i}}{\partial E_{m}}=-\frac{\mu}{2}-r_{m_{1}}p_{i}f_{i}$ |  |

Therefore, we can obtain these values:

|  | $r_{m_{1}}^{*}=\frac{\lambda_{i}E_{m}-(1-\lambda_{i})f_{i}}{(1-\lambda_{i})f_{i}u_{m}}$ |  |
| --- | --- | --- |
|  | $E_{m}^{*}=\frac{\sigma_{RC}-(1-\lambda_{i})}{(1-\lambda_{i})p_{i}}$ |  |

**Proof 10 for Lemma 5:**

Compared with the value $r_{m_{1}}^{*}$ under two different scenario 9 and 12, it can be seen that $E_{m}>\frac{(1-{\lambda_{i}}^{2})f_{i}}{\lambda_{i}(1+\lambda_{i}-2(1-\lambda_{i})u_{m})}$ when $\lambda_{i}\in(0,\frac{1}{2})$, $E_{m}<\frac{(1-{\lambda_{i}}^{2})f_{i}}{\lambda_{i}(1+\lambda_{i}-2(1-\lambda_{i})u_{m})}$ when $\lambda_{i}\in(\frac{1}{2},1)$. Compared with the value $E_{m}^{*}$ under two different scenario 9 and 12, it can be seen that $\lambda_{i}\in(0,\frac{2-\sigma_{RC}}{2})$ when $E_{m_{9}}^{*}>E_{m_{12}}^{*}$.Therefore, $E_{m}<\frac{(1-{\lambda_{i}}^{2})f_{i}}{\lambda_{i}(1+\lambda_{i}-2(1-\lambda_{i})u_{m})}$ always hold.

**Proof 11 for Policy Influencing Factors:**

1. The influence of Financial incentives

The optimal value $r_{m_{1}}^{*}$ of scenario 4 is $-\frac{1}{u_{m}}$, and the value of scenario 12 is $\frac{\lambda_{i}E_{m}-(1-\lambda_{i})f_{i}}{(1-\lambda_{i})f_{i}u_{m}}$. Simplify the $r_{m_{1}}^{*}$ of scenario 12, we obtained $\frac{\lambda_{i}(E_{m}-f_{i})+(1-f_{i})}{(1-\lambda_{i})f_{i}}\cdot(-\frac{1}{u_{m}})$. According to Assumptions, it can be seen that $\frac{\lambda_{i}(E_{m}-f_{i})+(1-f_{i})}{(1-\lambda_{i})f_{i}}<0$ and $\frac{\lambda_{i}(E_{m}-f_{i})+(1-f_{i})}{(1-\lambda_{i})f_{i}}\cdot(-\frac{1}{u_{m}})>0$.

2. The influence of Regulatory Penalties

The optimal value $r_{m_{1}}^{*}$ of scenario 4 is $-\frac{1}{u_{m}}$, and the value of scenario 12 is $\frac{\lambda_{i}E_{m}-(1-\lambda_{i})f_{i}}{(1-\lambda_{i})f_{i}u_{m}}$. Simplify the $r_{m_{1}}^{*}$ of scenario 12, we obtained $\frac{\lambda_{i}(E_{m}-f_{i})+(1-f_{i})}{(1-\lambda_{i})f_{i}}\cdot(-\frac{1}{u_{m}})$. According to Assumptions, it can be seen that $\frac{\lambda_{i}(E_{m}-f_{i})+(1-f_{i})}{(1-\lambda_{i})f_{i}}<0$ and $\frac{\lambda_{i}(E_{m}-f_{i})+(1-f_{i})}{(1-\lambda_{i})f_{i}}\cdot(-\frac{1}{u_{m}})>0$.

3. The influence of Environmental Benefits

According to Assumptions 5 and 6, $p_{i}\in\left( 0,1 \right).$Then, compared the value $E_{m}^{*}$ in this scenario with that in scenario 5 under the strict regulation mechanism, it can be seen that $E_{m_{9}}^{*}>E_{m_{5}}^{*}$ when $p_{i}\in\left( \frac{1}{f_{i}},1 \right)$.
